# Acalculous Cholecystitis Presenting as a Septic Joint: A Case Report

**DOI:** 10.7759/cureus.5193

**Published:** 2019-07-22

**Authors:** Yousaf Zafar, Ahmed A Elkafrawy, Julie Nahar, Muhammad Shafiq

**Affiliations:** 1 Internal Medicine, University of Missouri-Kansas City School of Medicine, Kansas City, USA; 2 Infectious Disease, University of Missouri-Kansas City School of Medicine, Kansas City, USA; 3 Internal Medicine, The University of Kansas Medical Center, Kansas City, USA

**Keywords:** septic joint, acalculous cholecystitis, clostridium perfringens

## Abstract

It is rare for acalculous cholecystitis to present with symptoms outside the abdomen; hence, making its diagnosis can be a challenge. We report a case of a 77-year-old male, with a relevant past medical history of left knee arthroplasty two years prior, who presented with left knee pain and swelling. Cultures from the arthrocentesis grew *Clostridium perfringens*, which led to a search for the source of infection. The right upper quadrant (RUQ) ultrasound (US) showed an enlarged gallbladder filled with sludge, but no cholelithiasis or secondary ultrasound findings were present to suggest acute cholecystitis. A computed tomography (CT) scan showed a distended gallbladder with diffuse gallbladder wall thickening and no stone but with suspicion for acalculous cholecystitis. A subsequent hepatobiliary (HIDA) scan confirmed the diagnosis of acalculous cholecystitis. Subsequently, the patient had a biliary drain placed. Bile cultures grew gram-positive rods consistent with *Clostridium perfringens*, confirming the source. With regards to the septic prosthetic joint, the patient underwent irrigation and debridement with polyethylene exchange without replacement of the prosthesis. The patient was also treated with six weeks of intravenous (IV) ertapenem (1 gram daily) and 12 months of moxifloxacin (400 mg daily). He had a cholecystectomy later and his symptoms were completely resolved.

## Introduction

Prosthetic joint infection (PJI) is a serious complication that occurs in 1% to 2% of total knee arthroplasties [1]. PJI results in significant morbidity and necessitates the replacement of the prosthetic joint in most cases. Typical organisms include *Staphylococcus aureus* and *Staphylococcus epidermidis* [2-3]. The route of infection is usually direct inoculation during surgery, contiguous spread from adjacent infection and sometimes from hematogenous spread [3]. Less commonly, gram-negative rods and *Enterococci* can also involve a prosthetic joint [4-5], but it is rare for *Clostridium perfringens* to cause infection of a prosthetic joint. It is challenging in such a clinical scenario to not only identify the source of infection (as the prosthetic knee joint or the surrounding skin/tissue is not where *Clostridium *species colonize) but also how to treat such PJI, as no guidelines exist to date.

## Case presentation

A 77-year-old Caucasian man presented with a three-day history of fever and left knee pain with swelling. His significant past medical history included diffuse large B-cell lymphoma (DLBCL) stage II-a in the sigmoid colon (for which he underwent a sigmoidectomy and chemotherapy and had been in remission for five years before presentation), chronic systolic heart failure secondary to Adriamycin, ventricular tachycardia with a subsequent implantable cardioverter defibrillator, and left total knee replacement two years before presentation. The patient denied any recent trauma or previous sexually transmitted diseases. He also denied any complications in his prosthetic joint after the surgery.

At the time of admission, the patient had a temperature of 102°F with a blood pressure of 100/75 mmHg and pulse of 87/minute and was maintaining 97% saturation on pulse oximetry on room air. Upon examination, he had diffuse swelling, erythema, and tenderness of the left knee. No other joints were involved, and the abdominal examination was unremarkable.

A radiograph of the left knee revealed joint effusion with stable implants. After a complete blood count, the patient was found to have a white blood cell count of 13,000/uL, hemoglobin of 15.5 g/dL, and platelet count of 144,000/uL. The patient’s comprehensive metabolic panel showed a sodium level of 134 mEq/L, potassium 3.9 mEq/L, blood urea nitrogen 52 mg/dL, creatinine 1.4 mg/dL, glucose 128 mg/dL, alanine aminotransferase 27 IU/L, aspartate aminotransferase 75 IU/L, and total bilirubin level of 0.9 IU/L. The patient’s inflammatory markers revealed an erythrocyte sedimentation rate of 32 mm/h and a C-reactive protein level of 269 mg/L. The patient’s lactic acid level was 1.4 mmol/L.

Arthrocentesis of the left knee joint revealed a cloudy, turbid synovial fluid with 141,530 leukocytes/uL, 86% of which were neutrophils. The gram stain of the synovial fluid reported gram-positive rods, which were later confirmed to be *Clostridium perfringens* on cultures.

The patient was diagnosed with septic arthritis and was started on IV vancomycin 1000 mg every 12 hours, IV ciprofloxacin 400 mg every 12 hours, and IV metronidazole 500 mg every eight hours. This antibiotic choice was based on the patient’s penicillin allergy. He underwent irrigation and debridement of the left knee, bone, and soft tissue with polyethylene tibial component exchange but without prosthetic joint removal. An operative anaerobic culture reported *Clostridium perfringens* on Day 3*. *Blood cultures and other synovial fluid cultures, including aerobic, fungal, and acid-fast bacilli (AFB) cultures were negative.

**Figure 1:**
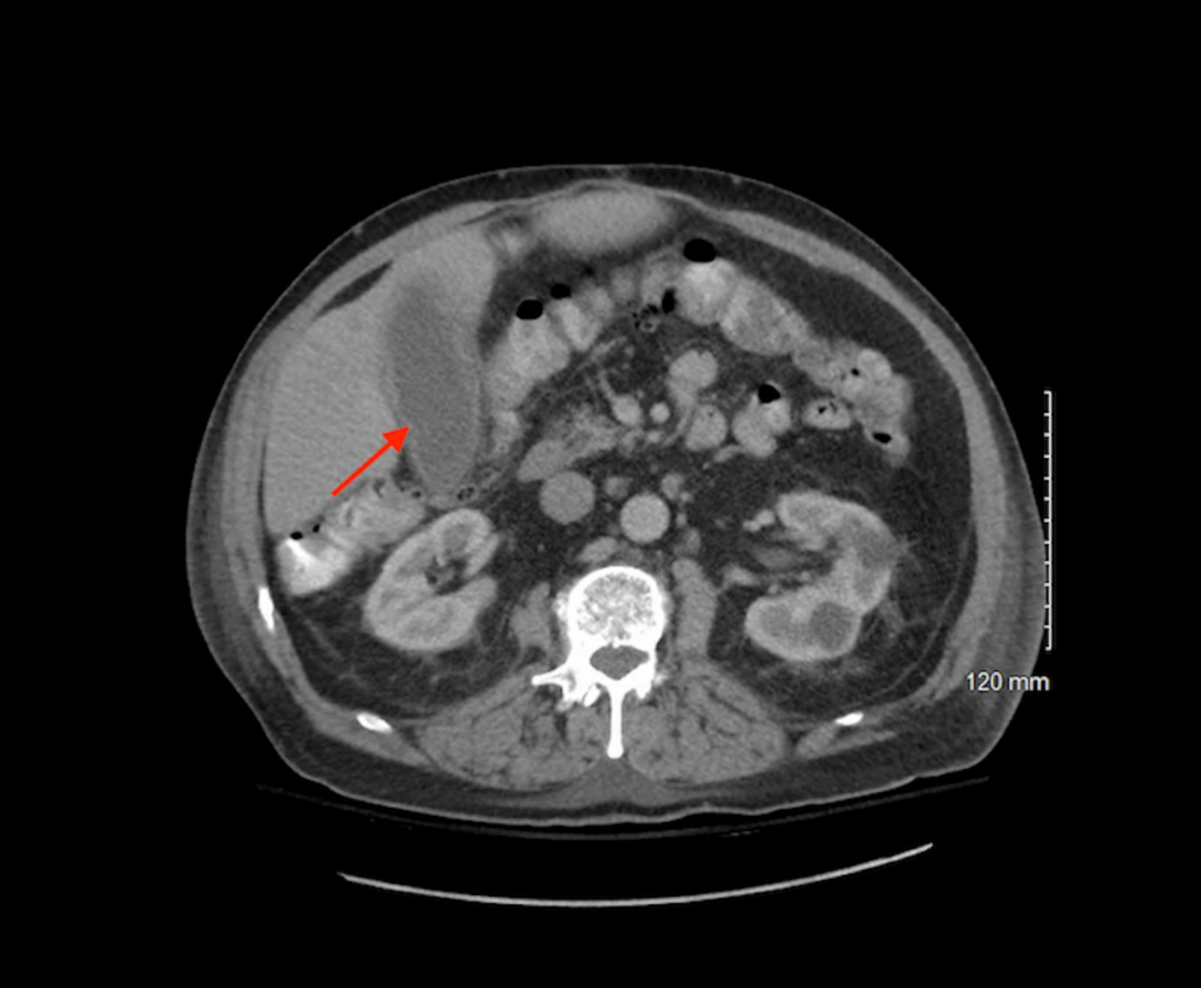
Computed tomography of the abdomen showing dilated gallbladder.

Even though the patient denied abdominal pain, nausea, vomiting, or diarrhea, given the rare incidence of a *Clostridium perfringens *prosthetic joint infection, a gastrointestinal source was suspected given his history of sigmoid colon DLBCL. An infectious disease team was consulted, and the patient’s antibiotics were switched to IV ertapenem 1 gram daily for activity against *Clostridium* along with IV moxifloxacin 400 mg daily for better penetration of the prosthetic joint. The RUQ US showed an enlarged gallbladder filled with sludge but no cholelithiasis or secondary ultrasound findings to suggest acute cholecystitis. A CT scan of the abdomen and pelvis with contrast reported a distended gallbladder with diffuse gallbladder wall thickening and with no stone but with suspicion for acalculous cholecystitis (Figure 1).

**Figure 2:**
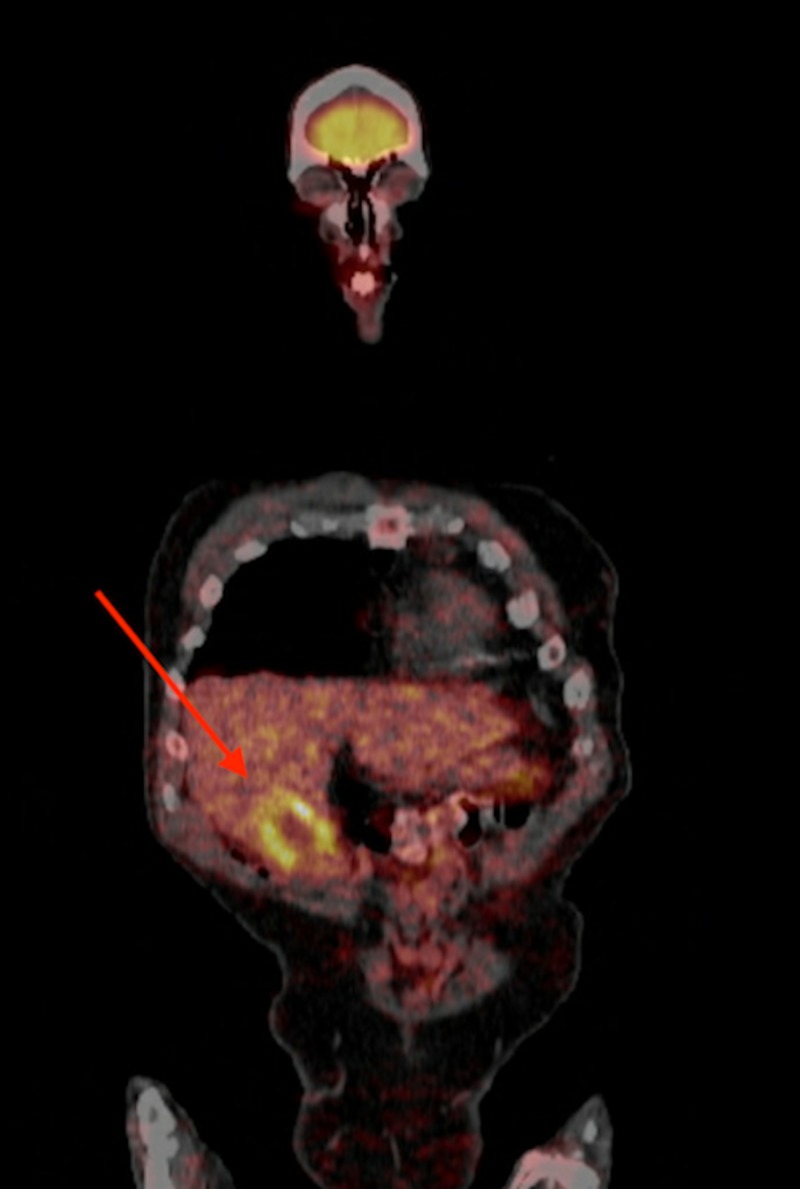
Positron emission tomography scan showing increased update around the gallbladder.

**Figure 3:**
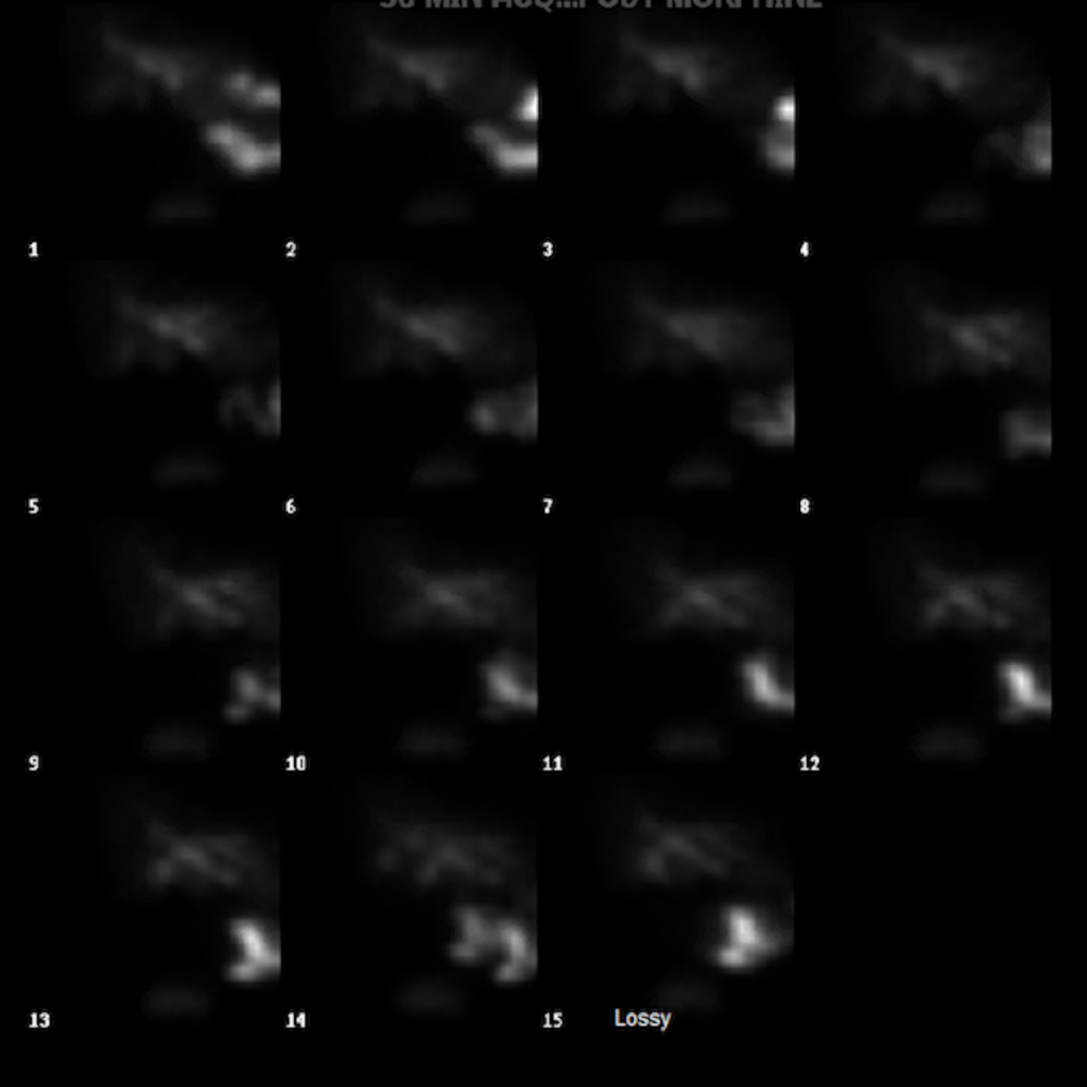
HIDA scan can showing no filling of the gallbladder. HIDA: hepatobiliary iminodiacetic acid

Subsequently, a positron emission tomography (PET) scan was performed to rule out the recurrence of lymphoma and reported a distended gallbladder with gallbladder wall increased fluorodeoxyglucose (FDG) uptake, which was concerning for acalculous cholecystitis as well ( Figure 2). Given the results of the CT scan of the abdomen and the increased FDG uptake on the PET scan, a hepatobiliary iminodiacetic acid (HIDA) scan was performed. The HIDA scan reported non-visualization of the gallbladder, which suggests acute cholecystitis or cystic duct obstruction (Figure 3).

An urgent biliary drain was placed, and the fluid drained was sent for analysis and cultures. Gram stains of the biliary fluid reported gram-positive rods suggestive of *Clostridium*. Final aerobic, anaerobic, fungal, and AFB cultures from the biliary drainage reported no growth.

A colonoscopy was performed to rule out the recurrence of lymphoma (which could have been the portal of entry to the *Clostridium* infection), and it showed three less than 5 mm polyps, which were resected. Pathology reports later stated hyperplastic polyps and a tubular adenoma. The patient’s clinical condition improved markedly with the antibiotic therapy, and he was discharged to an inpatient facility to complete IV ertapenem 1 gram for a total of six weeks. The patient’s IV moxifloxacin was switched to an oral formulation of the same dose (400 mg daily) that was prescribed for a total of 12 months. Laparoscopic cholecystectomy was performed about six weeks later, and the pathology results reported marked acute on chronic cholecystitis with associated hemorrhage. An intraoperative cholangiogram did not show any stones or biliary obstruction. The patient’s symptoms resolved completely, and at his six-month follow-up visit, he did not have any symptoms or signs suggestive of infection in the left knee prosthetic joint, and he was able to ambulate without any limitations.

## Discussion

*Clostridium perfringens* are obligate, in-anaerobes, gram-positive rods. They have been implicated in myonecrosis [6], cellulitis [7], and toxin-mediated food poising [8] and can even cause sepsis with or without associated hemolytic anemia [9]. However, it is rare for *Clostridium perfringens* to cause PJI, and it has been reported only in a few cases to date [10-12]. This rarity makes the treatment of PJI challenging, as there are no guidelines to date for its treatment.

Penicillin was reportedly effective in the previous cases, but our patient was effectively treated with IV ertapenem 1 gram daily for six weeks and moxifloxacin 400 mg daily for 12 months (the patient received it via IV initially while in the hospital but was switched to an oral formulation at the time of discharge), given his penicillin allergy.

We found only one case of *Clostridium perfringens* PJI that was caused by hematogenous spread from symptomatic acalculous cholecystitis, which was a complication directly after surgery [12]. Our case is unique because the acalculous cholecystitis was asymptomatic, and the infection occurred two years after the surgery.

Also, acalculous cholecystitis is a serious intra-abdominal infection that usually occurs in critically ill and immunosuppressed patients and has a high mortality if treatment is delayed [13]. A high index of suspicion is crucial for diagnosis, especially when the presentation is very nonspecific such as in our clinical scenario.

## Conclusions

*Clostridium perfringens* PJI is very uncommon and can occur by hematogenous spread from acalculous cholecystitis. Therefore, when the diagnosis of PJI by *Clostridium perfringens* is confirmed, a work-up for its source should be sought. In particular, a high index of suspicion is needed for asymptomatic cholecystitis in such patients, especially if the patient is immunocompromised and/or critically ill at the same time. Treatment is challenging, and further case reports and studies will help in developing guidelines in order to treat *Clostridium perfringens* PJI.
